# Urinary Bladder Hamartoma: Narrative Literature Review of an Exotic Pathology and Rare Cause of LUTS

**DOI:** 10.3390/clinpract15120218

**Published:** 2025-11-24

**Authors:** Mohammed Rafea Kanaan, Meryem Akkoyun, Marcel Lafos, Markus Antonius Kuczyk, Hossein Tezval

**Affiliations:** 1 Department of Urology and Urological Oncology, Hanover Medical School (MHH), 30625 Hanover, Germanytezval.hossein@mh-hannover.de (H.T.); 2 Department of Pathology, Hanover Medical School (MHH), 30625 Hanover, Germany

**Keywords:** hamartoma, urinary bladder, benign tumor, LUTS, case report

## Abstract

Urinary bladder hamartoma is an exceptionally rare benign lesion composed of disorganized yet mature tissue elements native to the bladder, including urothelium, fibrous stroma, smooth muscle, and occasionally adipose tissue. Unlike malignant tumors, it lacks cytological atypia, mitotic activity, or necrosis. Patients most often present with lower urinary tract symptoms (LUTS) or hematuria, though some cases are incidental findings. Associations with syndromic conditions such as Peutz–Jeghers, Beckwith–Wiedemann, Goldenhar, and Loeys–Dietz have been reported. Transurethral resection is the preferred treatment and has generally been curative. We report the first case in Germany—and the 16th worldwide—of urinary bladder hamartoma, occurring in a young adult male with bothersome LUTS. Because of its proximity to both ureteral orifices, only partial transurethral resection was performed, which provided durable symptom relief at 14 months of follow-up. This case highlights partial TUR as a pragmatic, organ-preserving alternative when complete resection is anatomically unsafe, while emphasizing that TURBT remains the standard of care. We provide a detailed discussion of the histopathological features, differential diagnosis, treatment considerations, and an updated narrative review of all reported cases.

## 1. Introduction

Hamartomas are abnormally arranged growths of normal cells indigenous to the organ, resulting in the formation of a mass or tumor [1,2]. They can manifest in various body parts, including the lungs, intestines, urinary bladder, skin, heart, brain, and breasts [1,3,4].

Although most hamartomas are asymptomatic, some can cause symptoms like painless hematuria, irritative voiding symptoms, or urinary retention if they grow sufficiently large [5]. Although the exact etiology remains unclear, a subset of hamartomas has been linked to congenital or genetic syndromes such as Peutz–Jeghers, Beckwith–Wiedemann, Goldenhar, and Loeys–Dietz, suggesting a possible developmental or germline predisposition [4,6,7,8]. The literature indicates that the tumor is rare, with only 15 published cases to date [4]. Notably, the rarity of this condition can lead to misdiagnosis. However, appropriate testing and diagnosis can result in proper treatment with positive outcomes.

Here, we report the case of a 22-year-old male with bladder hamartoma presenting with LUTS. In addition, we provide an updated narrative review of previously published cases.

## 2. Case Presentation

A 22-year-old man was admitted to our urology department with LUTS persisting for two months, significantly reducing his quality of life due to nocturia >3–4 times per night and pollakiuria during the day. A normal urinalysis with U-Stix ruled out urinary infection.

Ultrasonography of the bladder revealed an intravesical growth. Despite lacking typical risk factors (smoking, carcinogen exposure, or advanced age), the lesion’s cystoscopic features raised initial suspicion for urothelial carcinoma. However, urine cytology revealed no neoplastic cells.

**Figure 1:**
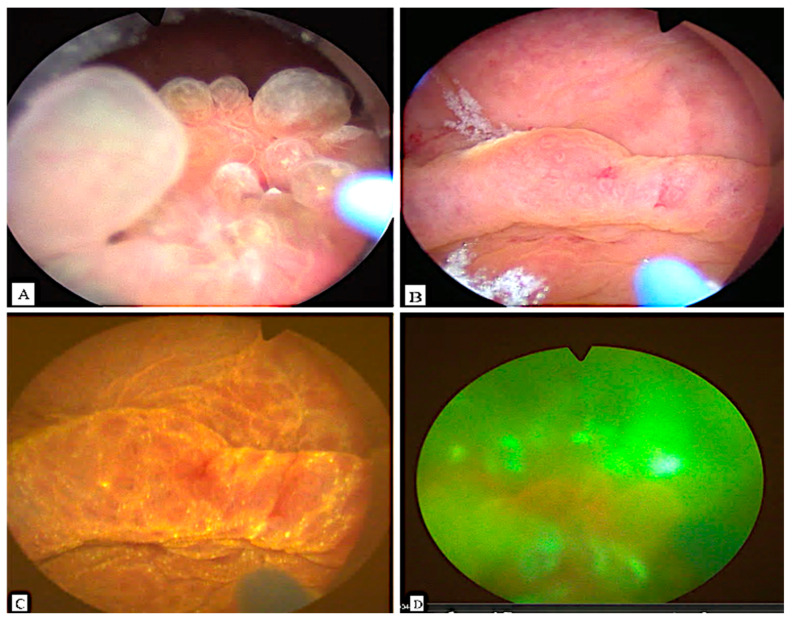
Transurethral images of the polypoid lesion in the urinary bladder under white light (**A**–**C**) and fluorescent cystoscopy with hexaminolevulinate with a negative signal (**D**).

Cystoscopy demonstrated a large mass between the trigone and the posterior bladder wall, closely localized to the ureteral ostia. Photodynamic diagnosis (PDD) with hexaminolevulinate was negative (Figure 1). A biopsy was performed in the initial session to exclude malignancy.

**Figure 2:**
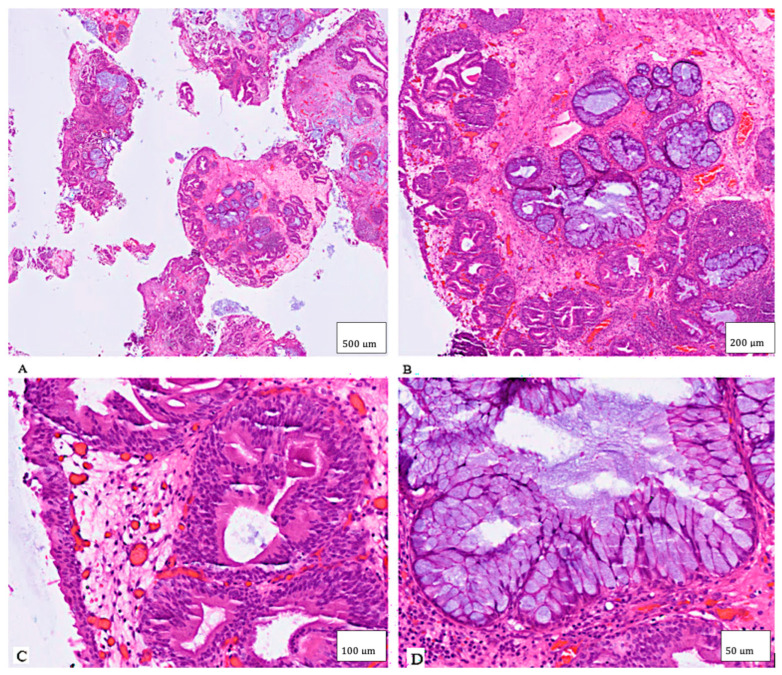
The lesion showed multiple fragmented tissue sections with irregularly shaped lobules and glandular structures within a fibro-muscular stroma ((**A**), H&E stain, 4× magnification, scale bar = 500 µm) ((**B**), H&E stain, 10× magnification, 200 µm) ((**C**), H&E stain, 20× magnification, 100 µm) ((**D**), H&E stain, 40× magnification, 50 µm). Abbreviations: H&E, hematoxylin and eosin.

Histopathological examination revealed that the tumor was a hamartoma of the urinary bladder with pronounced urocystitis cystica and glandularis of the intestinal type, in addition to extensive intestinal metaplasia, partially abundant in goblet cells, and focal mucin extravasation. No evidence of mitosis, necrosis, or atypical features was detected in either the epithelium or the stroma (Figure 2). Although immunohistochemistry was not performed in this case, prior reports have shown positivity for keratin 8/18, EMA, p63 and negativity for PAX8, CD10, AMACR, findings that help distinguish hamartomas from nephrogenic adenoma or urothelial carcinoma.

Based on the histopathological findings, a diagnosis of urinary bladder hamartoma was established. During the second transurethral session, a partial resection of the lesion was performed to achieve local control and to reduce the obstructive mass at the bladder outlet responsible for the patient’s lower urinary tract symptoms. Complete excision was not feasible because of the lesion’s close proximity to both ureteral orifices.

The patient experienced rapid and sustained symptom relief, reporting the absence of nocturia on the first day following Foley catheter removal. Genetic testing showed no evidence of syndromic or hereditary conditions associated with hamartomas. At 14-month follow-up, surveillance cystoscopy demonstrated stable residual tissue without regrowth or new symptoms, confirming a durable clinical response.

## 3. Literature Review

To contextualize our case, we performed a narrative literature review. The databases PubMed/MEDLINE, Google Scholar, and the Cochrane Library were searched up to January 2025. The following terms were used in different combinations: “urinary bladder hamartoma,” “hamartoma of the urinary tract,” and “bladder hamartoma”. The inclusion criteria were (i) peer-reviewed case reports or case series describing histologically confirmed hamartoma of the urinary bladder; (ii) English-language publications; and (iii) availability of clinical, pathological, or follow-up data. Exclusion criteria were (i) reviews, editorials, or conference abstracts without case-level data; (ii) animal studies; and (iii) reports without histopathological confirmation. 

Titles and abstracts were screened independently, and full texts were reviewed if relevant. Out of 92 initially identified publications, 15 met the criteria and were included in the qualitative synthesis. Our case was added as the 16th documented case worldwide. The synthesis is presented narratively and summarized in Table 1, which was reformatted and expanded by the authors to provide an original comparative overview.

The first urinary bladder hamartoma was reported by Lathan et al. in 1963, with gross hematuria and pyuria as the leading clinical manifestations [9]. A male predominance was observed in 11 of 16 cases (67%), and the mean age at diagnosis was 25 years [4].

The most common clinical presentation was irritative lower urinary tract symptoms (LUTS) with or without gross hematuria, occurring in 9 out of 16 cases (56%). Gross hematuria was present in 4 cases [4]. Remarkably, 31.2% of lesions (5/16) appeared on the background of specific syndromes, such as Peutz–Jeghers syndrome [6,10], Beckwith–Wiedemann syndrome [8], Goldenhar syndrome [7], and Loeys–Dietz syndrome (LDS) [4]. Other rare findings were associated with schistosomiasis (1/16) [11], prenatal detection via ultrasound (US) (1/16) [12], and incidental findings on abdominal imaging (1/16) [4].

The posterior bladder wall was the most commonly affected area (8/16, 50%), followed by the bladder neck (4/16, 25%), trigone (3/16, 19%), anterior wall (1/16, 6.2%), left lateral wall (1/16, 6.2%), and the bladder dome (1/16, 6.2%) [4].

Ota et al. and Pescia et al. examined urine cytology without significant alterations or dysplasias [3,4]. All the reported cases in the literature are summarized in Table 1, which also provides comprehensive data on urinary bladder hamartoma.

Cystoscopy was routinely performed across nearly all cases and served as a critical diagnostic and therapeutic modality.

Given their benign nature, transurethral resection of the bladder tumor (TURB) was the most commonly employed treatment modality and was curative in nearly all reported cases. In pediatric or congenital cases, partial cystectomy or en bloc resection via open or laparoscopic approaches was preferred, particularly when lesion size or location posed technical limitations to TURB. cystoscopic surveillance at 6 to 12 months post-resection, with no signs of recurrence or malignant transformation reported in the literature [4,6,8].

## 4. Discussion

Patients with urinary bladder hamartoma typically present with lower urinary tract symptoms (LUTS) or gross hematuria, although some cases are detected incidentally during imaging or cystoscopy. Reported cases span a wide age range, including several pediatric patients, and demonstrate a male predominance of approximately 67%. A subset of cases has been associated with syndromic conditions such as Peutz–Jeghers, Beckwith–Wiedemann, and Loeys–Dietz syndromes, suggesting a potential genetic or developmental predisposition. The current case aligns with these observations, involving a young male patient who presented with irritative LUTS, consistent with the most frequently reported clinical manifestation in the literature.

Imaging studies, including ultrasonography and computed tomography (CT), typically reveal a solid or polypoid mass, sometimes with central inhomogeneity, but cannot reliably distinguish hamartomas from malignant bladder tumors [4,15].

Cystoscopically, these lesions may resemble urothelial carcinoma, causing initial diagnostic uncertainty. A definitive diagnosis requires histopathological examination, which shows a lobulated, mixed proliferation of tubuloglandular and cystically dilated structures without atypia, embedded in fibromyxoid stroma with plump fibroblasts and increased vascularity. Immunohistochemistry may demonstrate positivity for keratin 8/18, EMA, and p63, and negativity for PAX8 and CD10; the absence of TERT promoter mutations further suggests a benign outcome [6]. Intestinal metaplasia and hypervascularity were also potential features observed in these lesions [3,5,6].

All patients in reported cases underwent successful treatment with complete excision via transurethral resection, partial cystectomy (3/16) [1,3,11], or transvaginal excision (1/16) [6]. Intravesical mitomycin was administered before histological examination because of the suspicion of malignancy [6]. In our case we choose to do a partial transurethral resection to avoid bigger damage, especially due to the proximity to the ostial region on both sides, as a new way to deal with these rare benign tumors.

No disease recurrence was observed, even after follow-up periods of up to 60 months through cystoscopy and ultrasound [4].

Possible differential diagnoses for urinary bladder hamartoma include cystitis cystica et glandularis, von Brunn hyperplasia, nested/microcystic variant of urothelial carcinoma, inverted urothelial papilloma, and nephrogenic adenoma, particularly in cases with unclear intravesical growth [13].

Distinctive features of urinary bladder hamartoma include its notably exophytic and lobulated structure, hypervascular stroma, and the presence of plump fibroblasts within disorganized, mature tissue components [17]. Differentiating it histologically from nephrogenic adenoma, a key mimic, is crucial since the latter exhibits specific immunohistochemical traits: usually positive staining for AMACR, CD10, and PAX8, and negative for p63 and CK20 [18,19]. Conversely, bladder hamartomas often lack a consistent immunoprofile but generally do not express these nephrogenic markers and have low Ki-67 proliferation indices, indicating their benign, non-proliferative nature [17].

One of the major diagnostic challenges in our case was distinguishing a urinary bladder hamartoma from other benign reactive or neoplastic entities. In particular, cystitis cystica et glandularis with a pseudo-polypoid configuration may closely resemble a hamartoma, given the presence of glandular structures and intestinal metaplasia. However, in our case, the architectural features were more consistent with a hamartomatous lesion: a lobulated, disorganized arrangement of mature epithelial and stromal elements, exophytic growth into the lumen, and the absence of cytological atypia, mitotic activity, or necrosis. We recognize that the absence of IHC reduces diagnostic certainty; however, the benign clinical course observed over 14 months of follow-up supports the diagnosis of a hamartoma. Future cases should include IHC and, when possible, molecular studies (such as TERT promoter mutation analysis) to increase diagnostic precision.

Nevertheless, its rarity and benign pathological features, which resemble cystitis cystica et glandularis or von Brunn nest hyperplasia, may mask its identification, potentially leading to underdiagnosis or misdiagnosis.

From a clinical perspective, most reported cases in the literature have been successfully treated with transurethral resection. However, in selected cases where complete resection is not feasible, close surveillance is essential to monitor potential progression or symptom recurrence. Given the benign nature of these lesions, aggressive interventions such as radical cystectomy should be avoided unless malignant transformation is confirmed. While transurethral resection remains the gold standard for bladder hamartomas and has been curative in nearly all reported cases, our case illustrates that partial resection may be a pragmatic choice in anatomically challenging situations, such as proximity to the ureteral orifices. This approach allowed symptom relief without risking iatrogenic damage.

Due to its rarity, the current body of evidence on urinary bladder hamartoma remains limited. Yet, the consistency of clinical presentation, radiological appearance, and histopathological characteristics across reported cases supports its recognition as a distinct benign urothelial entity. Importantly, its ability to mimic malignant bladder tumors, both clinically and endoscopically, highlights the critical role of comprehensive histopathological assessment—and, when appropriate, molecular analysis—to ensure accurate diagnosis and prevent unnecessary aggressive treatment.

Moreover, the recurring association with syndromic conditions in a subset of patients raises the possibility of a genetic or developmental etiology, warranting further investigation into underlying molecular pathways and germline mutations.

## 5. Limitations

This case has several limitations. First, no immunohistochemistry was performed, which would have provided additional evidence to differentiate hamartoma from mimickers such as nephrogenic adenoma or cystitis cystica/glandularis. Second, molecular testing (e.g., TERT promoter mutation analysis) was not available. Third, although we now report 14 months of follow-up, longer observation would be desirable to exclude late recurrence. Finally, as with all case reports, the conclusions drawn must be interpreted with caution, and our management approach (partial TUR) should not be generalized as a new treatment strategy but rather an individualized adaptation to a specific clinical context. Ongoing surveillance remains mandatory in such cases.

## 6. Conclusions

This report documents the first known case of urinary bladder hamartoma in Germany and the 16th case reported worldwide. Although complete transurethral resection (TURB) remains the standard treatment, partial resection may be a feasible and organ-preserving alternative in cases with difficult tumor locations, helping to avoid partial cystectomy. Since bladder hamartomas are benign, it is crucial to establish an accurate histopathological diagnosis to prevent overtreatment and guide proper clinical management. Increased awareness of this rare lesion, including its similarities with syndromic conditions and malignant mimickers, can help clinicians achieve timely and appropriate care.

## Figures and Tables

**Table 1:** Narrative Review of Urinary Bladder Hamartoma Published Cases to Date with our Case report (modified after Pescia et al. [4]).

Authors	Ref.	Y	Sex	Age	Localization	Clinical Presentation	Cytology	IHC	Therapy	Outcome	Follow-Up (Months)
Lathan & Garvey	[9]	1963	M	13	Left posterior wall with trigone extension	Gross hematuria and pyuria	NA	NA	TUR	No recurrence	60
Borski	[10]	1970	M	45	Bladder neck	LUTS	NA	NA	TUR	No recurrence	6
Keating et al.	[11]	1987	F	4	Posterior wall	Recurrent UTI in Peutz–Jeghers syndrome	NA	NA	Partial cystectomy	No recurrence	4
Park et al.	[12]	1989	F	45	Bladder dome	LUTS	NA	NA	TUR	NA	NA
Williams et al.	[8]	1990	M	0.8	Posterior wall	Hematuria in Beckwith–Wiedemann syndrome	NA	NA	TUR	No recurrence	18
McCallion et al.	[13]	1993	M	41	Trigone	LUTS with hematuria	NA	NA	TUR	No recurrence	60
Duvenage et al.	[14]	1997	M	19	Right posterior wall	Hematuria with schistosomiasis	NA	Muscle markers negative	TUR	No recurrence	5
Ota et al.	[3]	1999	F	58	Left posterior wall, invasive appearance on imaging	LUTS	No malignant cells	NA	TUR + partial cystectomy	No recurrence	36
Brancatelli et al.	[1]	1999	M	30	Right posterior wall, intramural	Gross hematuria, fever	NA	NA	Partial cystectomy	No recurrence	12
Adam et al.	[7]	2013	M	5	Trigone	LUTS with Goldenhar syndrome	NA	NA	TUR	No recurrence	2
Pieretti et al.	[15]	2014	M	0.2	Anterior wall	Prenatal detection	NA	S100−, HMB45−, keratin−, SMA+	TUR	No recurrence	18
Murray et al.	[5]	2015	F	51	Bladder neck	LUTS	NA	NA	Transvaginal excision	No recurrence	2
Al Shahwani et al.	[16]	2016	M	15	Left lateral wall	LUTS	NA	NA	TUR	No recurrence	NA
Kumar et al.	[6]	2021	M	20	Bladder neck	LUTS in Peutz–Jeghers syndrome	NA	NA	TUR + mitomycin	NA	NA
Pescia et al.	[4]	2022	F	54	Left posterior wall	Incidental finding	No malignant cells	keratin 8/18+, EMA+, p63+, keratin 7 focal+, PAX8−	TUR	NA	NA
Present case	NA	2023	M	22	Bladder neck	LUTS	No malignant cells	NA	Partial TUR	No recurrence of symptoms	14

Abbreviations: TUR: Transurethral resection; LUTS: Lower urinary tract symptoms; IHC: Immunohistochemistry; NA: Not assessed.

## Data Availability

Data supporting the findings of this study are available from the corresponding author upon reasonable request.
